# *Dracocephalum moldavica* L. Flavonoids Alleviate Doxorubicin-Induced Cardiotoxicity by Activating the AMPK/PGC1αPathway to Preserve Mitochondrial Homeostasis

**DOI:** 10.3390/ijms27135641

**Published:** 2026-06-23

**Authors:** Ruifang Zheng, Yanwen Du, Shoubao Wang, Wenling Su, Kaderyea Kader, Lijuan Zhang, Zihan Wang, Diwei Liu, Jianguo Xing, Shifeng Chu, Ming Xu

**Affiliations:** 1 School of Preclinical Medicine and Clinical Pharmacy, China Pharmaceutical University, Nanjing 211198, China; zrfangdd@163.com; 2 Xinjiang Key Laboratory of Uygur Medical Research, Xinjiang Institute of Materia Medica, Urumqi 830004, Chinayws990715@163.com (W.S.); kaderya0303@163.com (K.K.); 2229840722@stu.xjmu.edu.cn (L.Z.);; 3 Beijing Key Laboratory for Novel Drugs Discovery and Crystal Form Research in Cerebrovascular Diseases, Institute of Materia Medica, Chinese Academy of Medical Sciences & Peking Union Medical College, Beijing 100050, China

**Keywords:** total flavonoids of *Dracocephalum moldavica* L., doxorubicin-induced cardiotoxicity, mitochondrial damage, AMPK/PGC1α pathway, functional food

## Abstract

Doxorubicin (DOX) is a potent chemotherapeutic drug, whose clinical application is largely restricted by dose-dependent cardiotoxicity (DIC). *Dracocephalum moldavica* L. is a classic medicinal and edible plant with obvious cardiovascular protective effects; however, the role of its total flavonoids (TFDM) in DIC remains unclear. This study explored the cardioprotective effect of TFDM on DOX-induced myocardial injury and its mechanism related to mitochondrial quality control. We established in vivo and in vitro DIC models and adopted echocardiography, detection of cardiac injury and oxidative stress indicators, transmission electron microscopy, mitochondrial functional assessment and Western blotting, with AMPK knockdown performed for mechanism verification. Results showed that TFDM effectively improved cardiac function, reduced myocardial oxidative stress and apoptosis, and maintained mitochondrial ultrastructure and energy metabolism. TFDM activated the AMPK/PGC1α signaling axis to facilitate mitochondrial biogenesis, and AMPK silencing eliminated the protective effect of TFDM. In conclusion, AMPK/PGC-1α pathway is a primary key pathway involved in TFDM’s protective effects, which provides an experimental basis for the development of *Dracocephalum moldavica* L. as a functional food and adjuvant agent against DIC.

## 1. Introduction

Doxorubicin (DOX) is a widely used anthracycline chemotherapeutic agent with established efficacy in cancer treatment. However, its clinical application is substantially limited by dose-dependent cardiotoxicity [1], known as doxorubicin-induced cardiotoxicity (DIC), which can lead to severe cardiac dysfunction and even heart failure [2]. The pathogenesis of DIC is multifactorial and not fully elucidated, with mitochondrial dysfunction, oxidative stress, and inflammatory responses identified as key contributing mechanisms [3,4,5].

Among these, mitochondrial dysfunction has been recognized as a central event in DIC. DOX exhibits a high affinity for mitochondria, accumulating at concentrations up to 100-fold higher than in plasma [6]. It binds irreversibly to cardiolipin on the inner mitochondrial membrane, disrupting its function as an essential cofactor for respiratory chain enzymes [7]. This interaction impairs oxidative phosphorylation, reduces ATP production, and ultimately promotes cardiomyocyte death. Given the critical role of mitochondrial integrity in cardiomyocyte repair, targeting mitochondrial pathways has emerged as a promising therapeutic strategy to alleviate DIC [8]. Interventions aimed at preserving mitochondrial function may activate endogenous protective mechanisms and improve cardiac outcomes.

*Dracocephalum moldavica* L. is a classic medicinal plant commonly used in Uyghur and Mongolian traditional medicine to balance body fluids and nourish the heart [9]. Its application is documented in several traditional ethnic medical classics, representing over 700 years of clinical experience. It is believed to work by clearing endogenous heat toxins, thereby restoring the balance of qi-blood and body fluids in the heart meridians. This therapeutic effect is not only recorded in the ancient Uyghur medical canon Ali Kanun but also included in the National Drug Standard of the People’s Republic of China-Uyghur Medicine Volume [10,11,12]. To date, 29 individual flavonoid compounds have been isolated and identified from the active fraction of *Dracocephalum moldavica* L., of which the total content of six core flavonoid glycosides accounts for approximately 53%. These comprise tilianin (13.0–17.0%), acacetin-7-O-β-D-glucuronide (10.0–13.5%), rosmarinic acid (8.0–11.3%), luteolin-7-O-β-D-glucuronide (8.0–9.0%), diosmetin-7-O-β-D-glucuronide (7.0–8.6%), and apigenin-7-O-β-D-glucuronide (2.0–3.0%) [13]. Existing pharmacological evidence indicates that TFDM can inhibit the proliferation and migration of vascular smooth muscle cells, ameliorate H2O2-induced cardiotoxicity, and confer protection against myocardial ischemia/reperfusion injury and chronic heart failure [14,15]. Collectively, this evidence suggests that TFDM may also have potential in ameliorating DOX-induced cardiomyopathy.

In this study, we demonstrate that TFDM effectively alleviates DOX-induced cardiotoxicity by preserving cardiac contractility and mitigating mitochondrial dysfunction in cardiomyocytes. Mechanistically, TFDM reverses the DOX-triggered feedback suppression of AMPK phosphorylation, thereby activating the PGC-1α–NRF1–TFAM pathway and enhancing mitochondrial respiratory function. Our results highlight TFDM as a promising natural product candidate to prevent and treat doxorubicin-induced cardiotoxicity.

## 2. Results

### 2.1. TFDM Alleviates DOX-Induced Cardiac Systolic Dysfunction and Myocardial Injury

To investigate the protective effects in vivo, we first established a doxorubicin-induced cardiotoxicity (DIC) mouse model using a cumulative dose of 20 mg/kg DOX. The successful establishment of the DIC model was confirmed by a progressive and significant loss of body weight in DOX-treated mice (Figure S1a), alongside characteristic histopathological injuries in myocardial tissues, including disordered myofibrillar arrangement and cytoplasmic vacuolization (Figure S1b).

**Figure 1:**
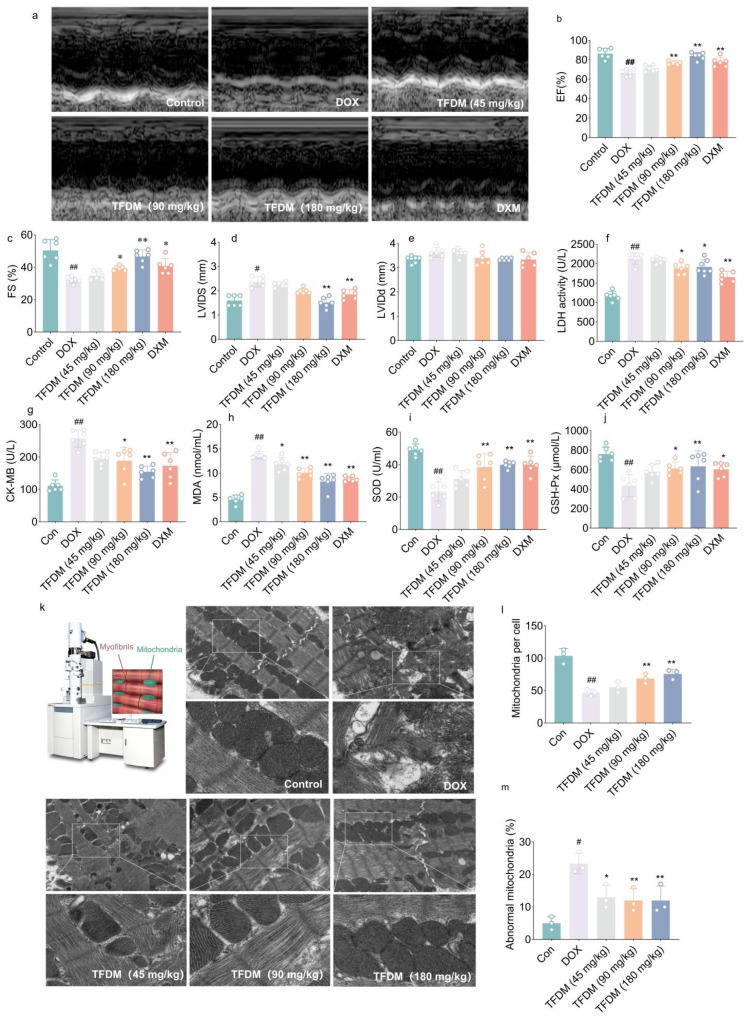
TFDM alleviated cardiac dysfunction and myocardial injury in mice subjected to DOX injection. (**a**) Representative images of M-mode echocardiograms from each group. (**b**–**e**) Ejection fraction (EF, (**b**)), fractional shortening (FS, (**c**)), left ventricular internal diameter at end-systole (LVIDs, (**d**)) and left ventricular internal diameter at end-diastole (LVIDd, (**e**)) were analyzed to assess their cardiac dysfunction (*n* = 6). (**f**,**g**) Activities of LDH (**f**) and CK-MB (**g**) in sera as cardiac damage markers. (*n* = 6). (**h**–**j**) Levels of MDA and activities of SOD and GSH-Px to reveal the oxidative stress in myocardial oxidative stress (*n* = 6). (**k**–**m**) Representative mitochondrial images from left ventricle myocardial tissue by TEM (The lower panel is the magnification from the white rectangle in the upper panel. magnification: upper panel 5000×; lower panel 30,000×), (**k**) and their analysis, including mitochondria number per cell (**l**) and the percentage of abnormal mitochondria (**m**) (*n* = 6). Data were shown as mean ± SD, # *p* < 0.05, ## *p* < 0.01 vs. the control group; * *p* < 0.05, ** *p* < 0.01 vs. the DOX group.

To evaluate the protective effects of TFDM against DIC, cardiac function was assessed by echocardiography on 15th day post TFDM administration. Echocardiographic analysis revealed significant cardiac dysfunction in C57BL/6J mice following DOX administration (Figure 1a), characterized by reduced left ventricular ejection fraction (EF, *p* < 0.01, Figure 1b) and fractional shortening (FS, *p* < 0.01, Figure 1c), while increased left ventricular internal diameter during systole (LVIDs, *p* < 0.05, Figure 1d). TFDM treatment at doses of 90, and 180 mg/kg significantly improved EF and FS compared to the DOX group (*p* < 0.05 at 90 mg/kg for FS; *p* < 0.01 at 90 mg/kg for EF and 180 mg/kg for both EF and FS, Figure 1b,c). Notably, the doses of 180 mg/kg effectively attenuated the DOX-induced increase in LVIDs (*p* < 0.05, Figure 1d), demonstrating TFDM’s capacity to preserve ventricular structure and systolic function. DXM, as the positive control agents also showed improvement on the cardiac function, confirmed the availability of this attenuation by TFDM. However, left ventricular internal diameter during diastole (LVIDd), an indicator of diastolic function, remained unchanged across all groups, suggesting that DOX primarily impaired systolic rather than diastolic function in this model (Figure 1e).

Assessment of serum biomarkers of confirmed these findings. DOX-treated mice exhibited significantly elevated levels of LDH and CK-MB, indicative of myocardial injury when compared to control group (*p* < 0.01 for both LDH and CM-KB, Figure 1f,g). TFDM administration substantially reduced the level of LDH at both doses of 90 and 180 mg/kg (*p* < 0.05, Figure 1f), while the contents of CM-KB showed remarkable attenuate at all of testes does (*p* < 0.05 at 90 mg/kg; *p* < 0.01 at 180 mg/kg, Figure 1g), suggesting a dose-dependent cardio protective effect.

In addition, TFDM mitigates oxidative stress in myocardial tissue. DOX administration induced pronounced oxidative stress, as evidenced by increased MDA levels and decreased activities of SOD and GSH-Px (*p* < 0.01, Figure 1h–j). TFDM treatment significantly reduced MDA levels at all doses (*p* < 0.05 at 45 mg/kg; *p* < 0.01 at 90 and 180 mg/kg, Figure 1h). The 90 mg/kg and 180 mg/kg doses notably elevated both SOD (*p* < 0.01 at both 90 and 180 mg/kg, Figure 1i) and GSH-Px (*p* < 0.05 at 90 mg/kg; *p* < 0.01 at 180 mg/kg, Figure 1j) activities, indicating restoration of antioxidant defenses. These findings demonstrate that TFDM exerts comprehensive cardioprotective effects against DOX-induced cardiotoxicity, especially for DOX-induced cardiomyopathy.

Ultrastructural analysis revealed that DOX caused severe mitochondrial damage, including reduced mitochondrial number per cell (*p* < 0.01, Figure 1k,l), swelling, and cristae fragmentation (*p* < 0.01, Figure 1k,m). TFDM treatment significantly improved mitochondrial morphology, increased mitochondrial number per cell at the dosage of 90 and 180 mg/kg (*p* < 0.01, Figure 1l). Notably, the mitochondrial morphology was attenuated at all tested dosages of TFDM, as presented as reduced percent of abnormal mitochondria (*p* < 0.05 at 45 mg/kg; *p* < 0.01 at 90 and 180 mg/kg, Figure 1m). Control mice exhibited normal myofibril organization and mitochondrial structure with intact cristae, while TFDM-treated groups showed marked preservation of these features compared to the DOX group. Collectively, TFDM alleviates DOX-induced cardiac systolic dysfunction and myocardial injury.

### 2.2. TFDM Mitigated DOX-Induced Cardiomyocyte Injury in H9c2 Cells

**Figure 2:**
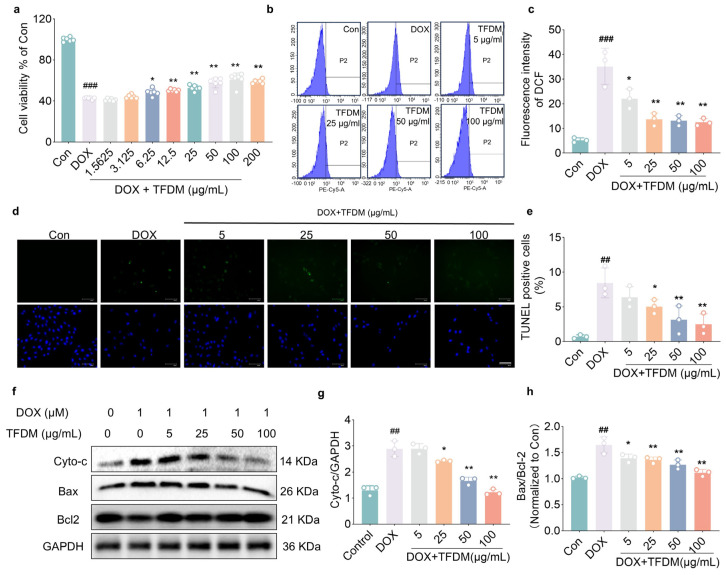
TFDM attenuates DOX-induced injury in H9c2 cardiomyocytes. (**a**) Cell viability was measured by CCK-8 assay. TFDM treatment concentration-dependently improved the viability of H9c2 cells exposed to DOX (*n* = 6). (**b**,**c**) Intracellular ROS levels were detected using the DCFH-DA probe and analyzed by flow cytometry. (**b**) Representative flow cytometry histograms; (**c**) Quantitative analysis of ROS fluorescence intensity (*n* = 3). (**d**,**e**) Apoptosis was evaluated by TUNEL staining. (**d**) Representative fluorescent images (scale bar: 100 μm); (**e**) Quantification of TUNEL-positive cells (*n* = 3). (**f**–**h**) Representative blots of Cyto-c, Bax and Bcl2 (**f**) and their analysis, including the levels of Cyto-c (**g**) and the ratio between Bax/Bcl-2 (**h**) (*n* = 3). Data were shown as mean ± SD. ## *p* < 0.01, ### *p* < 0.001 vs. control group; * *p* < 0.05, ** *p* < 0.01 vs. DOX group.

To directly evaluate the therapeutic potential of TFDM against DOX-induced cardiomyocyte injury, we utilized H9c2 cells as an in vitro model. Exposure to 1 μM DOX for 24 h significantly reduced cell viability to ~40% of control levels (*p* < 0.01, Figure 2a). Treatment with TFDM (6.25–200 μg/mL) dose-dependently restored cell viability (*p* < 0.05 at 6.25 μg/mL; *p* < 0.01 from 12.5 to 200 μg/mL, Figure 2a). Notably, the protective effect plateaued at 100 μg/mL, with no further improvement at 200 μg/mL Therefore, subsequent experiments were conducted using TFDM at concentrations ranging from 5 to 100 μg/mL.

DOX triggers oxidative damage in cardiomyocytes by generating free radicals via iron-mediated Fenton reactions. Consistent with in vivo findings in this study (Figure 1h), co-treatment with TFDM for 24 h alleviated oxidative stress in H9c2 cells. The DCFH-DA assay revealed a marked increase in ROS (measured by DCF fluorescence intensity) in DOX-treated cells (*p* < 0.001, Figure 2b,c). TFDM treatment significantly attenuated it, with ROS levels decreasing dose-dependently (*p* < 0.05 at 5 μg/mL; *p* < 0.01 at 25–100 μg/mL, Figure 2c).

TUNEL staining confirmed that DOX exposure markedly increased apoptosis (*p* < 0.01, Figure 2d,e). TFDM (25–100 μg/mL) significantly reduced apoptotic percentage (*p* < 0.01 at 25 μg/mL, *p* < 0.01 at 50 and 100 μg/mL). Mechanistically, DOX upregulated pro-apoptotic proteins (Bax, Cyto-c) and downregulated anti-apoptotic protein Bcl-2 (Figure 2f). TFDM treatment reversed these alterations, lowering the levels of Cyto-c (*p* < 0.05 at 25 μg/mL; *p* < 0.01 at 50 and 100 μg/mL, Figure 2g) and the ratio of Bax/Bcl-2 (*p* < 0.05 at 5 μg/mL; *p* < 0.01 at 25–100 μg/mL, Figure 2g), demonstrating its anti-apoptotic efficacy. Collectively, these findings demonstrate that TFDM against DOX-induced cardiomyocyte injury by reducing oxidative stress and apoptosis.

### 2.3. TFDM Ameliorates DOX-Induced Mitochondrial Dysfunction in H9c2 Cells

**Figure 3:**
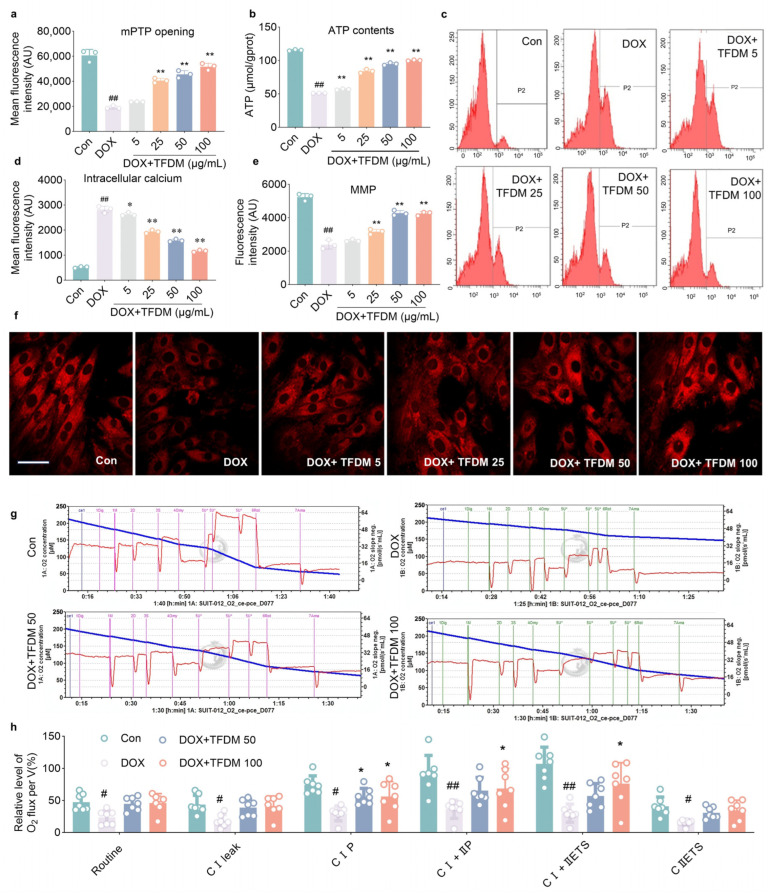
TFDM relieved DOX-caused mitochondrial abnormities in H9c2 cells. (**a**) mPTP opening was assessed using the BBcellProbe M61 kit. Fluorescence intensity is inversely proportional to the extent of mPTP opening (*n* = 3). (**b**) Intracellular ATP content was measured (*n* = 5). (**c**,**d**) Intracellular Ca^2+^ levels were determined by flow cytometry with Fluo-4 AM staining. (**c**) Representative histogram plots. (**d**) Quantitative analysis of fluorescence intensity (*n* = 3). (**e**,**f**) Mitochondrial membrane potential (ΔΨm) was evaluated using MitoTracker Red CMXRos. (**e**) Quantitative analysis of red fluorescence intensity. (**f**) Representative fluorescent images (scale bar: 10 μm) (*n* = 3). (**g**,**h**) Mitochondrial respiratory function was analyzed by high-resolution respirometry. (**g**) Representative oxygen consumption traces (* denotes replications of the U (FCCP) application). (**h**) Quantitative assessment of key mitochondrial respiratory parameters. The oxygen consumption rates were normalized to the total cell count (1 × 10^6^ cells per chamber) to ensure cross-sample comparison (*n* = 7). All data are expressed as mean ± SD. # *p* < 0.05, ## *p* < 0.01 vs. control group; * *p* < 0.05, ** *p* < 0.01 vs. DOX group.

To investigate the effects of TFDM on mitochondrial function in cardiomyocytes, we first assessed the opening of the mPTP, a critical regulator of mitochondrial integrity that orchestrates energy metabolism, redox balance, and cell fate. Using the BBcellProbe™ M61 fluorescent probe, we observed that DOX treatment significantly reduced fluorescence intensity (*p* < 0.01, Figure 3a), indicating enhanced mPTP opening. In contrast, TFDM (5, 25, 50, and 100 μg/mL) concentration-dependently restored fluorescence intensity (*p* < 0.01 at 25–100 μg/mL, Figure 3a), suggesting alleviation of mPTP opening induced by DOX in H9c2 cells.

The mPTP opening disrupts the proton gradient across the mitochondrial membrane, leading to reduced ATP synthesis (*p* < 0.01, Figure 3b) and loss of mitochondrial membrane potential (ΔΨm, *p* < 0.01, Figure 3e,f), which were all found in DOX treated cells as compared to controls. Notably, TFDM significantly reversed DOX-induced ΔΨm collapse (*p* < 0.01 at 25–100 μg/mL, Figure 3e) and restored ATP production (*p* < 0.05, Figure 3b), demonstrating its ability to alleviate mitochondrial energy metabolism dysfunction. Additionally, the enhanced mPTP opening promotes mitochondrial Ca^2+^ leakage, exacerbating cytosolic Ca^2+^ overload (*p* < 0.01, Figure 3c,d). Compared to the DOX group, TFDM significantly reduced intracellular Ca^2+^ levels (*p* < 0.05 at 5 μg/mL; *p* < 0.01 at 25–100 μg/mL, Figure 3d), suggesting improved calcium homeostasis.

To further evaluate mitochondrial respiratory function, we performed high-resolution respirometry (Figure 3g). In comparison to the control group, the DOX group exhibited a significant decrease across several key metabolic parameters, including routine respiration (*p* < 0.05), CI leak (*p* < 0.05), CI oxidative phosphorylation (CIP, *p* < 0.05), CI+IIP (*p* < 0.01), CII electron transfer system (CIIETS, *p* < 0.01) and CI+IIETS (*p* < 0.05, Figure 3h). These reductions indicated a marked impairment in mitochondrial function associated induced by DOX exposure, highlighting its impact on cellular energy metabolism. Both DOX+TFDM 50 μg/mL and DOX+TFDM 100 μg/mL groups showed notable elevation of CIP (*p* < 0.05), and treatment with 100 μg/mL TFDM significantly raised CI+IIP (*p* < 0.05) and CI+IIETS (*p* < 0.05), indicating improved mitochondrial respiratory function. Collectively, these findings demonstrate that TFDM effectively counteracts DOX-induced mitochondrial dysfunction in cardiomyocytes.

### 2.4. TFDM Counteract the DOX-Induced Feedback Inhibition of AMPK/PGC1α Signaling Pathway

Although the DOX-induced decline in ATP levels can trigger the energy-sensing kinase AMPK, leading to its transient activation, prolonged DOX exposure results in a burst of ROS and disruption of Ca^2+^ homeostasis. These effects cause oxidative modification of cysteine residues in AMPK, ultimately leading to its feedback inhibition [16,17]. This suppression subsequently blocks mitochondrial gene transcription, forming a vicious cycle [18].

**Figure 4:**
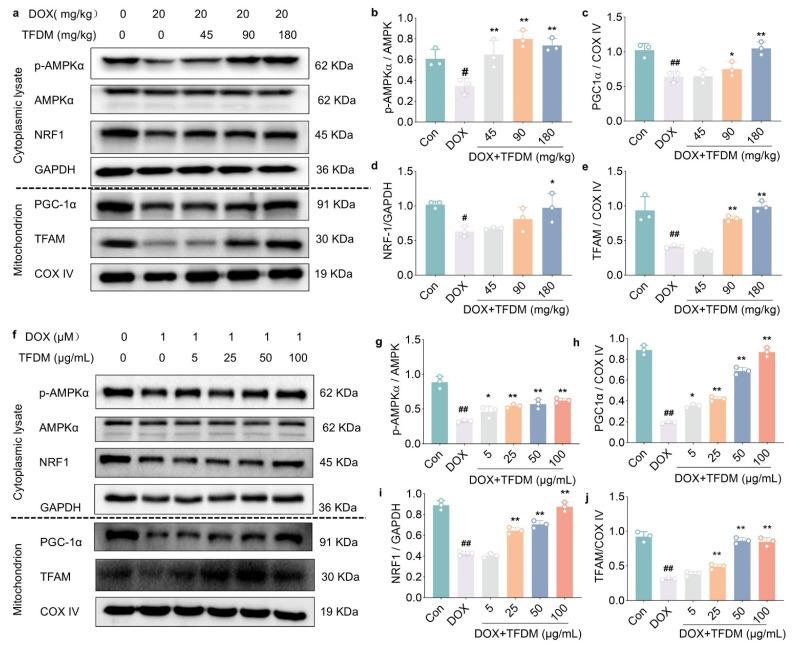
TFDM activates the AMPK/PGC1α signaling pathway suppressed by DOX. (**a**) Representative immunoblots of AMPKα, p-AMPKα and NRF1 in whole-cell lysates, and PGC1α, TFAM in mitochondrial fractions isolated from myocardial tissue. (**b**–**e**) Quantitative analysis of (**b**) the p-AMPKα/AMPKα ratio, (**c**) PGC1α, (**d**) NRF1, and (**e**) TFAM protein levels in myocardial tissue (*n* = 3). (**f**) Representative immunoblots of AMPKα, p-AMPKα and NRF1 in whole-cell lysates, and PGC1α, TFAM in mitochondrial fractions from H9c2 cells. (**g**–**j**) Quantitative analysis of (**g**) the p-AMPKα/AMPKα ratio, (**h**) PGC-1α, (**i**) NRF1, and (**j**) TFAM protein levels in H9c2 cells (*n* = 3). All data are expressed as mean ± SD. # *p* < 0.05, ## *p* < 0.01 vs. control group; * *p* < 0.05, ** *p* < 0.01 vs. DOX group.

To investigate the effect of TFDM on AMPK signaling, we examined the phosphorylation levels of AMPK in cardiac apical tissues. Immunoblot analysis revealed a significant reduction in phosphorylated AMPKα in cardiac tissues of the DOX group compared to the control group (*p* < 0.05, Figure 4a,b). Concurrent decreases were also observed in the expression of downstream proteins PGC1α (*p* < 0.01, Figure 4c), NRF1 (*p* < 0.05, Figure 4d), and TFAM (*p* < 0.01, Figure 4e) within the mitochondrial fraction of cardiac tissues. These results suggest a disruption of energy sensing and regulatory mechanisms in the heart following DOX treatment. Notably, TFDM administration at all tests doses effectively counteracted the reduction in AMPKα phosphorylation (*p* < 0.01, Figure 4b) and increased the protein levels of PGC1α at the dose of 90 and 180 mg/kg (*p* < 0.05 at 90 mg/kg, *p* < 0.01 at 180 mg/kg, Figure 4c). The level of NRF1 was attenuated at the dose of 180 mg/kg (*p* < 0.05, Figure 4d), and TFAM was improved at 90 and 180 mg/kg (*p* < 0.01, Figure 4e) in the mitochondrial fraction.

Moreover, a reduction in phosphorylated AMPKα was observed in H9c2 cells following DOX exposure for 24 h (*p* < 0.01, Figure 4f,g), suggesting potential feedback inhibition within the AMPK/PGC1α signaling pathway. Consistent with this hypothesis, mitochondrial levels of PGC1α, NRF1, and TFAM were significantly decreased (*p* < 0.01, Figure 4h–j). In contrast, notable upregulation of these proteins was detected in the TFDM treatment groups at 25–100 μg/mL (*p* < 0.01). Administration of 5 μg/mL TFDM also markedly elevated the expression of PGC1α (*p* < 0.05, Figure 4h) and phosphorylated AMPKα (*p* < 0.05, Figure 4g).

To confirm the mitochondrial biogenesis induced by TFDM, we measured the mitochondrial DNA (mtDNA) copy number (via qPCR) and Citrate Synthase (CS) activity, which is a highly validated and specific marker for mitochondrial mass. As shown in Figure S1, DOX exposure significantly decreased both the relative mtDNA copy number (the ratio of mitochondrial Cox1 to nuclear Actb) and CS activity in H9c2 cells. Consistent with our hypothesis, co-treatment with TFDM dose-dependently reversed these reductions, effectively restoring both the mtDNA copy number (Figure S2a) and CS activity (Figure S2b).

These results indicate that TFDM attenuates DOX-induced suppression of the AMPK/PGC1α pathway, thereby playing a critical role in mitigating the detrimental effects of DOX on mitochondrial biogenesis and cellular energy homeostasis.

### 2.5. Knockdown of AMPKα Blockade of the Beneficial Effects of TFDM in DOX-Insulted H9c2 Cells

**Figure 5:**
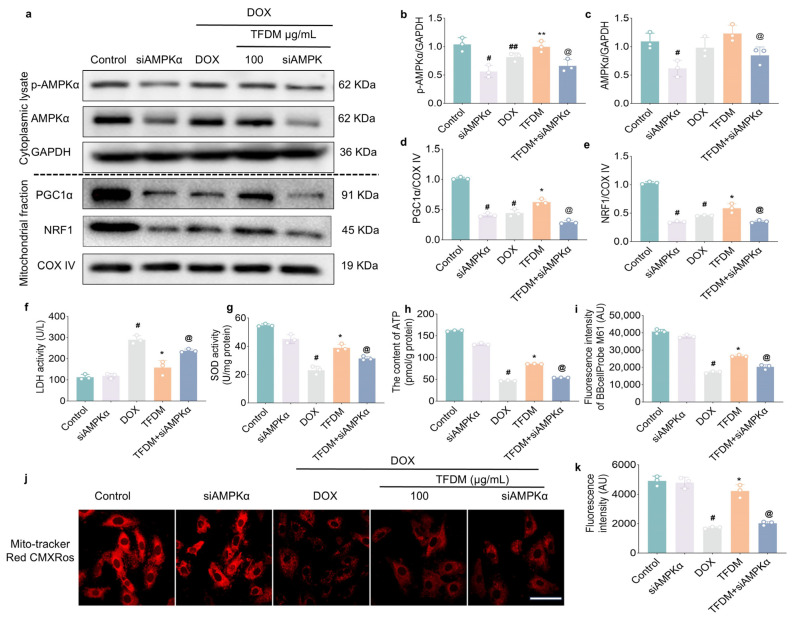
Knockdown of AMPKα abolishes the protective effects of TFDM in DOX-insulted H9c2 cells. (**a**) Representative immunoblots of AMPKα, p-AMPKα and PGC1αin whole-cell lysates, and NRF1 in mitochondrial fractions from H9c2 cells. (**b**–**e**) Quantitative analysis of (**b**) p-AMPKα, (**c**) AMPKα, (**d**) PGC-1α, (**e**) NRF1 protein levels (*n* = 3). (**f**,**g**) Activities of LDH (**f**) and SOD (**g**). (*n* = 3). (**h**) Intracellular ATP levels measured by Elisa. (*n* = 3). (**i**) mPTP opening was assessed using the BBcellProbe M61 kit. Fluorescence intensity is inversely proportional to the extent of mPTP opening (*n* = 3). (**j**,**k**) Mitochondrial membrane potential was evaluated using MitoTracker Red CMXRos. (**j**) Representative fluorescent images (scale bar: 8 μm). (**k**) Quantitative analysis of red fluorescence intensity. (*n* = 3). Data were shown as mean ± SD, # *p* < 0.05, ## *p* < 0.01 vs. control group; * *p* < 0.05, ** *p* < 0.01 vs. DOX group; @ *p* < 0.01 vs. TFDM group.

To investigate the role of AMPKα in the protective effect of TFDM against DIC, it was knocked down in H9c2 cells using siRNA, as AMPKα is known to activate PGC1α. Immunoblot analysis confirmed that transfection with AMPKα siRNA significantly reduced both AMPKα protein (*p* < 0.05, Figure 5a,c) and phosphorylated AMPKα levels (*p* < 0.05, Figure 5b) compared with control cells. This downregulation led to a marked decrease in the mitochondrial levels of PGC1α (*p* < 0.05, Figure 5d) and its downstream effector NRF1 (*p* < 0.05, Figure 5e), indicating effective blockade of the AMPK/PGC1α signaling pathway in H9c2 cells. Under these conditions, the beneficial effects of TFDM on enhancing mitochondrial PGC1α (*p* < 0.05, Figure 5d) and NRF1 (*p* < 0.05, Figure 5e) expression were abolished when AMPKα was knocked down, compared to the DOX+TFDM group.

We further evaluated the role of AMPKα in TFDM-mediated alleviation of oxidative stress and improvement in cell viability. Results showed that knockdown of AMPKα attenuated the ability of TFDM to suppress LDH release (*p* < 0.05, Figure 5f) and enhance SOD activity (*p* < 0.05, Figure 5g), compared to the DOX+TFDM group. In terms of mitochondrial function, silencing AMPKα counteracted the protective effects of TFDM on promoting ATP synthesis (*p* < 0.05, Figure 5h), inhibiting mPTP opening (*p* < 0.05, Figure 5i), and restoring mitochondrial membrane potential (*p* < 0.05, Figure 5j,k), relative to the DOX+TFDM treatment group.

Collectively, these findings indicate that AMPKα knockdown diminishes the beneficial effects of TFDM against DOX-induced mitochondrial dysfunction, underscoring the essential role of the AMPK/PGC1α signaling pathway in the protective mechanism of TFDM in mitigating DIC.

### 2.6. Pharmacological Inhibition of AMPK Abolishes the Protective Effects of TFDM in DOX-Insulted H9c2 Cells

**Figure 6:**
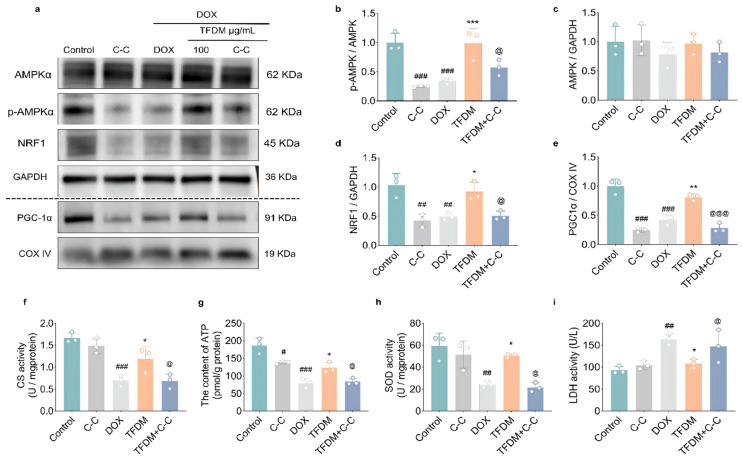
Pharmacological inhibition of AMPK abolishes the protective effects of TFDM in DOX-treated H9c2 cells. (**a**) Representative immunoblots of AMPKα, p-AMPKα, and NRF1 (with GAPDH as the cytosol/total loading control), and PGC-1α (with COX IV as the mitochondrial loading control) in H9c2 cells. (**b**–**e**) Quantitative analysis of (**b**) p-AMPKα/AMPKα, (**c**) AMPKα/GAPDH, (**d**) NRF1/GAPDH, and (**e**) PGC-1α/COX IV protein levels (*n* = 3). (**f**,**g**) Quantitative analysis of (**f**) CS activity and (**g**) intracellular ATP content (*n* = 3). (**h**,**i**) Intracellular SOD activity (**h**) and extracellular LDH release (**i**) in H9c2 cells (*n* = 3). Data are presented as mean ± SD. # *p* < 0.05, ## *p* < 0.01, ### *p* < 0.001 vs. control group; * *p* < 0.05, ** *p* < 0.01, *** *p* < 0.001 vs. DOX group; @ *p* < 0.01, @@@ *p* < 0.001 vs. TFDM group.

To further substantiate the requirement of AMPK activation in the protective mechanism of TFDM, we performed pharmacological rescue experiments using Compound C (C-C), a selective AMPK inhibitor. Immunoblotting confirmed that C-C treatment successfully blocked AMPK activation, as evidenced by a dramatic decrease in phosphorylated AMPKα (p-AMPKα) levels (*p* < 0.001, Figure 6a,b) without altering total AMPKα expression (Figure 6a,c). Consequently, the downstream nuclear transcription factor NRF1 (*p* < 0.01, Figure 6a,d) and mitochondrial PGC-1α (*p* < 0.001, Figure 6a,e) were significantly downregulated, demonstrating the successful blockade of the AMPK/PGC-1αsignaling axis. Under these conditions, the TFDM-mediated restoration of NRF1 (*p* < 0.05, Figure 6a,d) and PGC-1α (*p* < 0.001, Figure 6a,e) expression was significantly abrogated.

Consistently, the rescue effects of TFDM on mitochondrial function and cellular integrity were largely abolished by AMPK inhibition. The restoration of CS activity (*p* < 0.05, Figure 6f) and ATP production (*p* < 0.05, Figure 6g) by TFDM was significantly blocked by C-C. Moreover, C-C treatment attenuated the capacity of TFDM to boost SOD activity (*p* < 0.05, Figure 6h) and suppress LDH release (*p* < 0.05, Figure 6i) under DOX exposure. These pharmacological findings are consistent with our AMPKα gene silencing data, collectively demonstrating that TFDM alleviates DOX-induced cardiotoxicity in an AMPK-dependent manner.

## 3. Discussion

**Figure 7:**
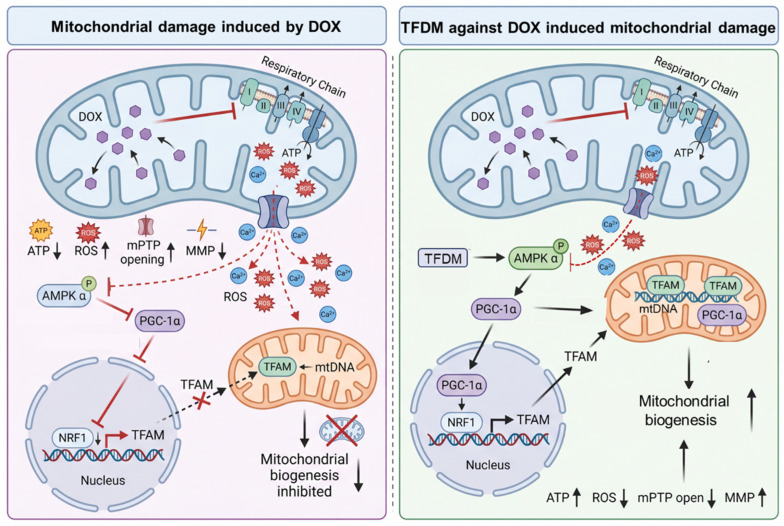
This Elevated ROS induced by DOX contribute to mitochondrial dysfunction by depolarizing the MMP, inducing mPTP opening, and impairing respiratory function, collectively leading to reduced ATP synthesis. Meanwhile, increased ROS, along with cytosolic Ca^2+^ overload, induce feedback inhibition of AMPKα, a key kinase normally activated under energy deficit, thus disrupting its role in promoting mitochondrial biogenesis. This suppression further compromises the mitochondrial repair capacity, creating a vicious cycle of deterioration. TFDM counteracts this process by restoring AMPK activity and enhancing the expression of mitochondrial biogenesis-related genes (e.g., PGC-1α, NRF1, TFAM). Consequently, TFDM alleviates DOX-induced mitochondrial injury by inhibiting mPTP opening, stabilizing MMP, improving respiratory function, and ultimately increasing ATP production, thereby preserving cardiomyocyte contractility and mitigating cardiotoxicity.

This study demonstrates that TFDM confers significant cardioprotection against DIC, primarily through the preservation of mitochondrial structure and function by activating the AMPK/PGC1α signaling axis (Figure 7). DOX cardiotoxicity is intimately linked to its profound impact on cardiac mitochondria. Our findings are consistent with the established model wherein DOX preferentially accumulates in mitochondria, intercalating into membrane phospholipids such as cardiolipin and disrupting the integrity of the inner mitochondrial membrane [19]. This structural compromise manifests as cristae rupture, organellar swelling, and a net loss of mitochondrial numbers, thereby undermining the fundamental infrastructure for energy production [20]. In this study, TFDM treatment effectively countered these structural aberrations, resulting in a higher mitochondrial count and a marked reduction in the proportion of abnormal organelles. This suggests that TFDM likely stabilizes mitochondrial membranes, conferring resistance to DOX-induced disintegration.

Beyond structural damage, DOX instigates severe functional deficits. A pivotal event is the induction of persistent mPTP opening. The irreversible opening of the mPTP dissipates the proton motive force, collapsing the ΔΨm, halting ATP synthesis, and triggering the mitochondrial apoptotic pathway via cytochrome c release [21]. Our data show that TFDM potently inhibits DOX-induced mPTP opening. By preventing this critical event, TFDM effectively maintained ΔΨm, thereby ensuring oxidative phosphorylation continuity and blocking apoptosis initiation.

Concurrently, DOX undergoes redox cycling, generating a surge of ROS that overwhelms endogenous antioxidant defenses, as evidenced by suppressed SOD and GSH-Px activities, and it also promotes lipid peroxidation [22]. This oxidative insult coincides with a severe impairment of mitochondrial respiratory function, leading to diminished oxygen consumption rates and a critical cellular energy deficit. TFDM exhibited dual antioxidant and bioenergetic restorative properties. It significantly reduced intracellular ROS accumulation and enhanced the activities of key antioxidant enzymes, thereby mitigating oxidative damage. Simultaneously, TFDM improved mitochondrial respiratory capacity, as reflected by the recovery of key respiratory parameters, ultimately restoring intracellular ATP levels and directly addressing the bioenergetic crisis induced by DOX. Notably, ROS levels in this study were primarily assessed using the DCFH-DA probe, which reflects the overall intracellular oxidative burden rather than mitochondria-specific ROS production. Therefore, the reduction in global ROS, together with preserved mitochondrial respiration, stabilized mitochondrial membrane potential and improved ultrastructural integrity, supporting the conclusion that TFDM alleviates mitochondrial oxidative injury. However, future studies using mitochondria-targeted ROS probes are warranted to further clarify its compartment-specific antioxidant mechanisms.

Notably, in evaluating oxidative stress, this study primarily utilized the DCFH-DA probe to monitor global intracellular ROS levels. While this probe effectively captures the overall cellular oxidative burst—a major hallmark of DOX cardiotoxicity—it is relatively non-specific and cannot distinguish between different cellular compartments, such as mitochondrial-derived ROS (mtROS). Nevertheless, the dramatic reduction in the overall cellular ROS pool, combined with our comprehensive evidence of preserved mitochondrial respiration (O_2_ flux), stabilized membrane potential, and intact ultrastructure, strongly indicates the amelioration of mitochondrial-derived oxidative damage. Future investigations utilizing mitochondria-targeted ROS probes (such as MitoSOX Red) are warranted to further delineate the precise, compartmentalized antioxidant mechanisms of TFDM.

The AMPK/PGC1α pathway is a central regulator of mitochondrial homeostasis and energy metabolism. While acute DOX exposure may transiently activate AMPK as a compensatory stress response to acute energy deficits, the literature indicates context-dependent outcomes [23,24,25]. Our mechanistic investigations align with the concept that chronic DOX treatment leads to excessive bursts of ROS and calcium dysregulation, which causes AMPK oxidative modification and ultimately results in the feedback inhibition of this pathway. This suppression downregulates the master regulators of mitochondrial biogenesis, PGC1α, NRF1, and TFAM, thereby impairing the replacement of damaged mitochondria and exacerbating dysfunction. A key finding of this study is that TFDM counteracted the DOX-induced suppression of AMPK phosphorylation, consequently reactivating the downstream PGC-1α–NRF1–TFAM axis. This transcriptional activation promotes the synthesis of proteins essential for mitochondrial biogenesis and function, facilitating the regeneration of a healthy mitochondrial network and sustaining long-term energetic homeostasis.

To unequivocally validate the essential role of AMPK in TFDM-mediated cardioprotection, we employed a dual-validation strategy combining genetic knockdown of the AMPKα subunit (via siRNA) and pharmacological inhibition (using C-C). While genetic silencing via siRNA provides highly specific, direct evidence by ablating AMPKα expression, the complementary use of the pharmacological inhibitor Compound C further strengthens our conclusions through a robust rescue paradigm. Crucially, both independent experimental approaches yielded highly consistent results: either AMPK genetic ablation or chemical blockade thoroughly abolished TFDM’s beneficial effects in doxorubicin-insulted H9c2 cells. Specifically, the loss of AMPK function completely abrogated the TFDM-induced upregulation of downstream effectors, NRF1, and mitochondrial PGC-1α and consequently prevented the recovery of mitochondrial function, as evidenced by the failure to restore ATP production, citrate synthase (CS) activity, and mitochondrial membrane potential (ΔΨm). Furthermore, the capacity of TFDM to suppress oxidative stress (SOD activity) and cellular injury (LDH release) was largely lost under these conditions.

In summary, our findings delineate a mechanism whereby the AMPK/PGC-1α pathway is a primary key pathway involved in TFDM’s protective effects, which in turn coordinates the improvement of mitochondrial integrity, attenuation of oxidative stress, restoration of bioenergetic capacity, and inhibition of apoptotic signaling. These results position TFDM as a promising therapeutic candidate for combating DIC. Therefore, these findings may provide new insights for developing functional foods from natural products (TFDM) that can prevent anthracycline chemotherapy-induced cardiotoxicity and exert cardioprotective effects in cancer patients.

### Limitations

Despite these promising findings, this study has several limitations. First, the in vitro experiments were primarily conducted on H9c2 myoblasts, which do not fully replicate the physiological features of mature adult cardiomyocytes. Future studies employing primary adult cardiomyocytes or human-induced pluripotent stem cell-derived cardiomyocytes are warranted to further validate the translational potential of TFDM. Second, while our current findings establish the crucial role of the AMPK/PGC-1α pathway in TFDM-mediated mitochondrial protection, it is essential to recognize that plant-derived flavonoids and polyphenols frequently exhibit pleiotropic pharmacological activities. In biological systems, mitochondrial homeostasis is governed by a highly integrated network rather than a single linear cascade. Notably, SIRT1, an NAD+-dependent deacetylase, is a pivotal co-regulator that works in close synergy with AMPK to orchestrate mitochondrial quality control. Further investigations utilizing SIRT1-specific inhibitors and evaluating PGC-1α deacetylation states are highly warranted to fully map the multi-target synergistic network of TFDM against DIC.

## 4. Materials and Methods

### 4.1. Chemicals and Agents

TFDM were produced by Xinjiang Key Laboratory of Uygur Medical Research, Xinjiang Institute of Materia Medica, and their main chemical compound were characterized as previously described [26]. Doxorubicin was purchased from Sigma-Aldrich (44583, Saint Louis, MA, USA).

### 4.2. Animal Experiments and Ethics Statement

Male C57BL/6 mice, 8-week-old, were obtained from SPF Biotechnology Co., Ltd. (Beijing, China) and fed with commercial food pellets and tap water under controlled environmental conditions maintained at a temperature of 24 ± 2 °C, humidity of 45% ± 5%, and a 12 h light-dark cycle. This research was conducted in accordance with the internationally accepted principles for laboratory animal use and care as found in the European Community guidelines (EEC Directive of 1986; 86/609/EEC). The protocol was reviewed and approved by the Animal Care and Use Committee of Xinjiang Institute of Materia Medica (No. XJIMM-2019003-6).

### 4.3. Establishment of DIC Model in Mice

The DIC mouse model was established according to the previously described method with slightly modifications [27], Mice were assigned at random into five groups: control group (Con), DOX injection group, DOX injection combined with TFDM treatment at doses of 45, 90 and 180 mg/kg/d, respectively. Con and DOX group mice were administered 0.5% sodium carboxymethylcellulose (CMC-Na, SJ-MB0055, SparkJade, Jinan, China) by oral gavage, while TFDM treated groups were administered respective amounts of TFDM dissolved in 0.5% CMC-Na daily for fourteen days. After seven days of TFDM pre-treatment, 20 mg/kg DOX was intraperitoneally injected twice with an interval of three-day. On the 15th day, mice were anesthetized and cardiac function was assessed via echocardiography, Subsequently, blood samples (0.5 mL) and cardiac tissues were collected following enucleation. Serum was separated from whole blood, and both serum and cardiac tissues were stored at −80 °C for further investigation.

### 4.4. Echocardiography

On the 15th day of the experiment, the animals were placed under light anesthesia (maintained with 1.5% isoflurane, R510-22-2, RWD Life science, Shenzhen, China) and underwent echocardiography using a Vevo 2100 imaging system (Medtronic Medical Electronic Co., Ltd., Shenzhen, China) in both B-mode and M-mode. Left ventricular internal dimensions at end-diastole (LVIDd) and end-systole (LVIDs) were measured from the echocardiographic images. These values were subsequently used to calculate ejection fraction (EF) and fractional shortening (FS) as indicators of cardiac function.

### 4.5. Transmission Electron Microscopy (TEM)

Freshly dissected mouse heart tissues underwent two washes with pre-cooled phosphate-buffered saline (PBS, P1038, Solarbio, Beijing China). Specimens were cut into 1–2 mm^3^ cubes and fixed in buffered glutaraldehyde (R31038, OriLeaf, Shanghai, China) for 24 h. Following dehydration, embedding in resin and staining, ultrathin sections were ready for imaging on a JEM-1230 TEM (JEOL Tokyo, Japan). For quantitative analysis, at least 10 random non-overlapping fields were evaluated per sample by an investigator blinded to the experimental groups. Mitochondria were classified as ‘abnormal’ if they exhibited notable swelling, severe vacuolization, or partial/complete loss of cristae.

### 4.6. Determination of MDA Level and Activities of LDH, CK-MB, GSH-Px and SOD

Whole blood samples collected from treated animals were allowed to clot naturally at ambient temperature and then centrifuged at 3000 rpm for 20 min. The resulting serum was used to measure the activities of lactate dehydrogenase (LDH; A020-2-2, Nanjing Jiancheng Biotechnology Co., Ltd., Nanjing, China) and creatine kinase-MB isoenzyme (CK-MB; E-EL-M0355, Elabscience, Wuhan, China). In addition, the level of malondialdehyde (MDA; E-EL-0060, Elabscience, Wuhan, China) and the activities of glutathione peroxidase (GSH-Px; E-BC-K096-M, Elabscience, Wuhan, China) and superoxide dismutase (SOD; A001-3-2, Nanjing Jiancheng Biotechnology Co., Ltd., Nanjing, China) in myocardial tissue were determined following the manufacturers’ kit instructions.

### 4.7. DIC Modeling and Cell Viability Assay In Vitro

H9c2 rat cardiomyocytes were obtained from the Cell Resource Center of the Institute of Basic Medical Sciences, Chinese Academy of Medical Sciences. The cells were maintained in low-glucose DMEM (31600-10, Solarbio, Beijing China) supplemented with 10% fetal bovine serum (FBS, 10099141C, Gibco, New York, NY, USA) and cultured at 37 °C in a 5% CO_2_ atmosphere. Upon reaching approximately 90% confluence, the cells were detached using a 0.25% trypsin solution containing EDTA (40127ES60, Yeasen, Shanghai, China) and subcultured. All experiments were performed during the logarithmic growth phase. The cultured H9c2 cells were randomly divided into six groups: a control group, a DOX-treated group, and four TFDM-treated groups (with TFDM concentrations ranging from 1.5625 to 200 μg/mL). Following a 6 h pretreatment with either vehicle or TFDM, the cells were exposed to 1 μM DOX for 24 h to establish an in vitro model of DIC. Cell viability was subsequently assessed using the CCK-8 assay (TP1197-3, Targetmol, Shanghai, China).

### 4.8. ROS Assay with DCFH-DA

Cells were collected by digestion and centrifuged, and all culture supernatants were discarded. After conducting two washes with PBS buffer, the solution was subsequently replaced with 2 μM DCFH-DA (ID3130, Solarbio, Beijing China) in PBS buffer. The samples were then incubated for 20 min at 37 °C while being shielded from light. Following incubation in complete DMEM (R28175, OriLeaf, Shanghai, China) for 10 min. and filtering with a 300-mesh sieve, the fluorescent intensity of 2′,7′-dichlorofluorescein (DCF) was analyzed on a BD FACSCalibur flow cytometer (Franklin Lakes, NJ, USA).

### 4.9. TUNEL Assay

Apoptosis was examined using a fluorescent terminal-deoxynucleotidyl transferase-mediated dUTP nick-end labeling (TUNEL) assay kit (C1170M, Beyotime, Shanghai, China). Briefly, cells underwent fixation in 4% paraformaldehyde and then permeabilization for 2 min on ice. Following TUNEL staining and DAPI counterstaining, the image was caught on a fluorescence microscope and analyzed with the ImageJ software (1.54g) to calculate the percentage of TUNEL-positive cells.

### 4.10. Determination of Intracellular Ca^2+^ Content

H9c2 Cell pellets following two PBS washes were incubated with 4 μΜ Fluo-4 AM (HY-101896, MCE, Shanghai, China) for 20 min at 37 °C under protection from light. Following 3 washes with chilled HEPES buffer (P011461, KonoScience, Beijing, China) and a 300-mesh sieve filtering, the fluorescence intensity was analyzed using a BD FACSCalibur flow cytometer.

### 4.11. Determination of Intracellular ATP Content

H9c2 cells were ultrasonically broken on ice. ATP content was detected using a commercial kit (S0026, Beyotime Biotechnology, Shanghai, China) based on the firefly luciferase bioluminescence method. The luminescence intensity, which is strictly proportional to the ATP concentration, was measured using a microplate reader and quantified against a standard curve.

### 4.12. Measurement of mPTP Opening

Mitochondria permeability transition pore (mPTP) opening was assessed using BBcellProbe M61 kit (BB-48122, BestBio, Shanghai, China) as reported previously [28]. The fluorescence intensity of BBcellProbe M61 probe was measured on a microplate reader (TECAN SPARK, Männedorf, Switzerland).

### 4.13. Mitochondrial Membrane Potential Measurement

We examined changes of mitochondrial membrane potential (MMP) using a MitoTracker CMXRos probe (C1999S, Beyotime Biotechnology, Shanghai, China) as previously described [29]. The fluorescence intensity was observed on a Nikon A1 confocal microscope.

### 4.14. Mitochondrial Respiratory Function Detection

The substrate-uncoupler-inhibitor titrations were used to detect mitochondrial respiratory function in permeabilized H9c2 cells on an Oroboros Oxygraph-O2k (Tissue Gnostics, Innsbruck, Austria) as described previously [14]. The oxygen consumption rates were normalized to the total cell count (1 × 10^6^ cells per chamber) to ensure accurate cross-sample comparisons. *n* = 7 independent biological replicates.

### 4.15. Western Blotting

Myocardial tissue and H9c2 cells were maintained on ice for protein extraction. Mitochondrial fraction and cytoplasmic fraction were prepared, respectively, by Mitochondria-Cytosol Isolation Kit (C1206, Applygen, Beijing, China). The BCA method was employed to assess protein amounts. Following gel electrophoresis and transfer, PVDF membranes (ISEQ00010, Millipore, Burlington, MA, USA) containing proteins were subjected to blocking with 5% fat-free milk (D8341, Solarbio, Beijing, China). The membranes were then incubated overnight with antibodies specific to the target proteins including AMPKα (2603S, 1:1000), p-AMPKα (2531L, 1:1000), Bax (36030S, 1:1000), Bcl-2 (28150S, 1:1000), Cytochrome c (11940T, 1:1000) (Cell Signaling Technology, Danvers, MA, USA), PGC1α (ab77210, 1:1000), TFAM (ab307302, 1:1000) and NRF1 (ab175932) (Abcam, Waltham, MA, USA) at 4 °C. Subsequently, the membranes were treated with horseradish peroxidase-conjugated secondary antibodies for two hours at room temperature. An enhanced chemiluminescence kit was utilized for detection, and ImageJ (1.54g) was employed for quantitation.

### 4.16. RNA Interference

AMPK alpha1/2 siRNA (sc-45313) and scrambled siRNA (sc-37007) were obtained from Santa Cruz Biotechnology (Dallas, TX, USA). When H9c2 cells in a 6-well plate grew to the convergence rate arriving at 70–80%, siRNA transfection using Lipofectamine RNAiMAX reagent (13778100, Invitrogen, Carlsbad, CA, USA) was carried out. The final concentration of siRNA used in this work was 10 nM. After 48 h of transfection, the cells were grouped into scrambled siRNA transfection (including Control, DOX, DOX+TFDM 100 μg/mL) and siAMPKα transfection (including siAMPKα, DOX + TFDM + siAMPKα).

### 4.17. Analysis of the Mitochondrial DNA Copy Number

H9c2 cells were seeded in 6-well plates at a density of 4 × 10^5^ cells/well and cultured in an incubator at 37 °C for 24 h. The cells were then exposed to various concentrations of TFDM with DOX (1 μM) for 24 h. Genomic DNA was extracted from the H9c2 cells using the EasyPure^®^ Genomic DNA Kit (EE101–01, TransGen Biotech, Beijing, China) according to the manufacturer’s instructions. DNA was amplified using an Applied Biosystems 7900HT real-time quantitative PCR system with the PerfectStart Green qPCR Supermix Kit (TransGen Biotech, AQ601–04, Beijing, China). The mitochondrial DNA copy number was measured as the relative ratio of mitochondrial DNA to nuclear DNA. The mitochondrial DNA level was evaluated by amplifying the mt-*Co1/Cox1* gene encoded on the heavy strand using the forward primer 5′-CCACTTCGCCATCATATTCGTAGG-3′ and the reverse primer 5′-TCTGAGTAGCGTCGTGGTATTCC-3′, while *Actb* was used as the nuclear DNA control with the forward primer 5′-GCAGGAGTACGATGAGTCCG-3′ and the reverse primer 5′-ACGCAGCTCAGTAACAGTCC-3′. The results were calculated using the 2^−ΔΔCT^ method.

### 4.18. Analysis of Citrate Synthase (CS) Activity

The activity of CS in H9c2 cells was determined using a Citrate Synthase (CS) Activity Assay Kit (BC1060, Solarbio, Beijing, China). H9c2 cells were seeded in 6-well plates at a density of 4 × 10^5^ cells/well and cultured for 24 h. Following exposure to various concentrations of TFDM with DOX (1 μM) for 24 h, the cells were collected and lysed. The CS activity of the cell lysates was then determined according to the manufacturer’s instructions.

### 4.19. Pharmacological Inhibition of AMPK

To pharmacologically inhibit AMPK, H9c2 cells were pretreated with the specific AMPK inhibitor Compound C (C-C, T1977, Targetmol, Shanghai, China, 5 μM) for 2 h prior to exposure to DOX (1 μM) and TFDM (100 μg/mL) for 24 h. Cell lysates, mitochondrial fractions, and supernatants were then collected for Western blot analysis, ATP content determination, CS activity, SOD activity, and LDH release assays as described above.

### 4.20. Statistical Analysis

Data analysis was conducted using a *t*-test for two-group comparisons and one-way analysis of variance (ANOVA) with subsequent Dunnett’s post hoc test for multiple group comparisons. Prior to ANOVA, the Shapiro–Wilk test was performed to verify data normality, and Levene’s test was used to assess the homogeneity of variances. The ‘n’ values specified in figure legends represent independent biological replicates. The statistical analyses were performed utilizing GraphPad Prism 8, and results are presented as mean ± standard deviation (SD). *p* < 0.05 was consider a significant difference.

## 5. Conclusions

In conclusion, this study systematically verified that the total flavonoids of *Dracocephalum moldavica* L. (TFDM) exert significant cardioprotective effects against DIC in both in vivo mouse models and in vitro H9c2 cardiomyocytes. TFDM effectively alleviated DOX-triggered cardiac systolic dysfunction, reduced myocardial oxidative stress and cardiomyocyte apoptosis, and preserved myocardial structural integrity. Mechanistically, TFDM targeted mitochondrial homeostasis by inhibiting mPTP opening, stabilizing mitochondrial membrane potential (MMP), restoring ATP production and improving mitochondrial respiratory function, thereby reversing DOX-induced mitochondrial damage.

Notably, TFDM exerted its protective role by activating the AMPK/PGC1α signaling pathway—it reversed the DOX-induced feedback inhibition of AMPK phosphorylation, upregulated the expression of downstream mitochondrial biogenesis-related proteins PGC1α, NRF1, and TFAM, and promoted mitochondrial biosynthesis. Knockdown of AMPKα using siRNA or pharmacological inhibition of AMPK activity abolished the mitochondrial protective nature of TFDM, confirming that the AMPK/PGC-1α pathway is a primary key pathway involved in TFDM’s protective effects.

Collectively, these findings demonstrate that TFDM ameliorates DOX-induced cardiotoxicity by regulating mitochondrial homeostasis. This study provides robust experimental evidence for TFDM as a promising natural cardioprotective agent and functional food component, offering a potential adjuvant therapeutic strategy to reduce the clinical cardiotoxic risk of DOX chemotherapy.

## Data Availability

The original contributions presented in this study are included in the article/Supplementary Materials. Further inquiries can be directed to the corresponding authors.

## Supplementary Materials

The following supporting information can be downloaded at: https://www.mdpi.com/article/10.3390/ijms27135641/s1.
