# Supplementary material for: Dracocephalum moldavica L. Flavonoids Alleviate Doxorubicin-Induced Cardiotoxicity by Activating the AMPK/PGC1αPathway to Preserve Mitochondrial Homeostasis
Source: Int J Mol Sci. 2026 Jun 23;27(13):5641. doi: 10.3390/ijms27135641 (PMC13361811; doi:10.3390/ijms27135641)
Supplement: Supplementary file 1 [file ijms-27-05641-s001.zip › ijms-4346351-Supplement information.pdf]

Supplement information

Dracocephalum moldavica L. Flavonoids Alleviate Doxorubicin-Induced Cardiotoxicity by Activating the AMPK/PGC1αPathway to Preserve Mitochondrial Homeostasis

Ruifang Zheng [1,2,†], Yanwen Du [2,†], Shoubao Wang [3], Wenling Su [2], Kaderyea Kader [2], Lijuan Zhang [2], Zihan Wang [3], Diwei Liu [2], Jianguo Xing [2], Shifeng Chu [2,3,*] and Ming Xu [1,*]

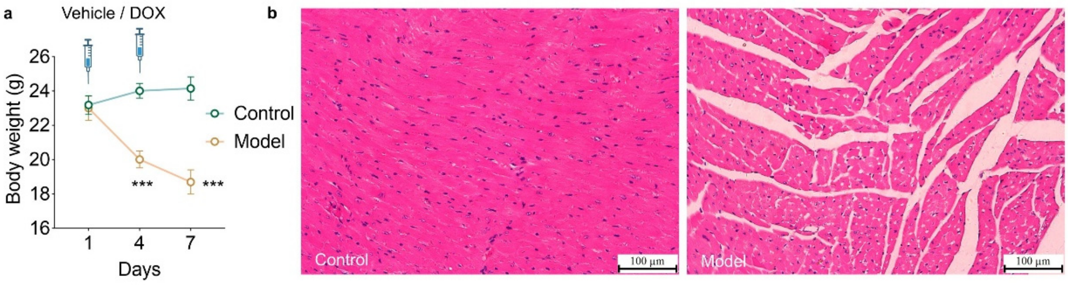

Figure S1. Validation of the DOX-induced cardiotoxicity (DIC) model in mice. (a) Body weight changes in mice after DOX treatment (n = 6). A cumulative dose of 20 mg/kg DOX was administered via two intraperitoneal (i.p.) injections with a 3-day interval. (b) Representative H&E staining images of cardiac sections from the Control group and DOX-treated Model group on day 7. Scale bar = 100 μm. Data are presented as Mean ± SD. ***P < 0.001 vs. Control group.

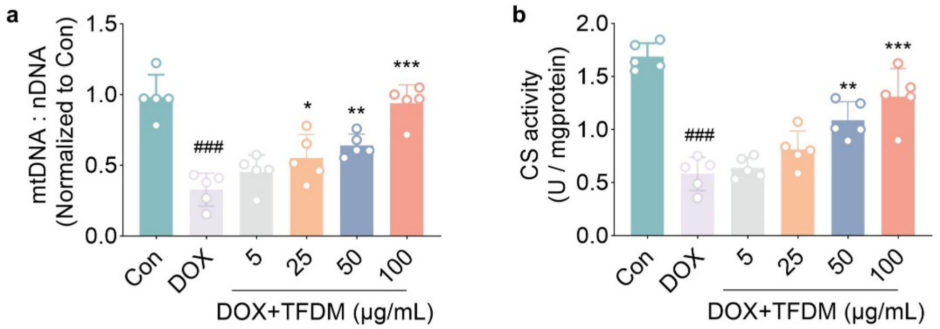

Figure S2. TFDM alleviates the loss of mitochondrial biogenesis induced by DOX in H9c2 cells. (a) TFDM protects against the reduction of mtDNA copy number induced by DOX. The levels of mtDNA were evaluated by the ratio between *Cox1* and *Actb*, representing mitochondrial DNA and nuclear DNA, respectively. (b) TFDM restores the CS activity reduced by DOX. n=5. All data are expressed as mean ± SD. ### P < 0.001 vs. the control group; * P < 0.05, ** P < 0.01, *** P < 0.001 vs. the DOX group.
